# Health-related quality of life with encorafenib plus binimetinib for *BRAF*^V600E^ thyroid cancer

**DOI:** 10.1530/ETJ-25-0273

**Published:** 2026-04-03

**Authors:** Naomi Kiyota, Makoto Tahara, Hiroo Imai, Shunji Takahashi, Akihiro Nishiyama, Shingo Tamura, Yasushi Shimizu, Shigenori Kadowaki, Ken-ichi Ito, Yoshinori Hirashima, Shinji Ueno, Iwao Sugitani

**Affiliations:** ^1^ Department of Medical Oncology and Hematology, Cancer Center, Kobe University Hospital, Kobe, Hyogo, Japan; ^2^ Department of Head and Neck Medical Oncology, National Cancer Center Hospital East, Kashiwa, Chiba, Japan; ^3^ Department of Medical Oncology, Tohoku University Hospital, Sendai, Miyagi, Japan; ^4^ Department of Medical Oncology, The Cancer Institute Hospital of JFCR, Koto-ku, Tokyo, Japan; ^5^ Department of Medical Oncology, Kanazawa University Hospital, Kanazawa, Ishikawa, Japan; ^6^ Department of Medical Oncology, NHO Kyushu Medical Center, Fukuoka, Fukuoka, Japan; ^7^ Department of Medical Oncology, Hokkaido University Hospital, Sapporo, Hokkaido, Japan; ^8^ Department of Clinical Oncology, Aichi Cancer Center Hospital, Nagoya, Aichi, Japan; ^9^ Division of Breast and Endocrine Surgery, Department of Surgery, Shinshu University School of Medicine, Matsumoto, Nagano, Japan; ^10^ ONO Pharmaceutical Co., Ltd., Osaka, Osaka, Japan; ^11^ Department of Endocrine Surgery, Nippon Medical School Hospital, Kashiwa, Chiba, Japan

**Keywords:** thyroid cancer, differentiated thyroid cancer, anaplastic thyroid cancer, health-related quality of life, patient-reported outcome, multitargeted tyrosine kinase inhibitors (MKIs), BRAF V600E, a BRAF inhibitor, a MEK inhibitor

## Abstract

**Background:**

A Japanese phase 2 trial of encorafenib plus binimetinib met the primary endpoint of the centrally assessed objective response rate in patients with unresectable *BRAF* V600E-mutated thyroid cancer. Consequently, encorafenib plus binimetinib has been approved in Japan. We present the health-related quality of life (HR-QoL) outcomes from the trial.

**Methods:**

The multicenter, open-label, uncontrolled phase 2 trial enrolled patients with unresectable locally advanced or metastatic *BRAF* V600E-mutated thyroid cancer. HR-QoL score, one of the secondary endpoints, was assessed using the EORTC QLQ-C30 and QLQ-THY34 at baseline and during follow-up. The minimal important change (MIC) threshold was set at 10 points.

**Results:**

We enrolled 22 patients with *BRAF* V600E-mutated thyroid cancer. The median follow-up was 11.5 months. Completion rates for the questionnaires were 100% (22 of 22 patients) at baseline and 68% (15 of 22) at week 20. Until week 20, changes from baseline were below MIC at most time points in 29 of all 32 domains in QLQ-C30 and QLQ-THY34. In the other 3 domains, patients showed tendencies toward improvement (social support and appetite loss) or deterioration (joint pain) for consecutive time points. Patients with anaplastic thyroid cancer showed tendencies toward improvement for swallowing, restlessness, body image, and discomfort in the head and neck, from the first assessment after the initial treatment.

**Conclusion:**

The combination therapy of encorafenib plus binimetinib for unresectable *BRAF* V600E-mutated thyroid cancer was associated with generally maintained HR-QoL. Considering the efficacy and safety data from the trial, the regimen may provide clinical benefits while maintaining HR-QoL.

## Introduction

Thyroid cancers include several histological types, such as papillary thyroid cancer (PTC) and follicular thyroid cancer, both of which are classified as differentiated thyroid cancer (DTC), as well as anaplastic thyroid cancer (ATC). DTC is a major subtype accounting for >90% of thyroid cancer cases. DTC progresses slowly and often requires long-term therapies. On the other hand, ATC is rare (1–2% of thyroid cancer) but progresses more rapidly, and the prognosis is often poor (1, 2, 3).

The driver mutation *BRAF* V600E is often detected in thyroid cancer. Comprehensive genomic profiling reveals that the *BRAF* V600E mutation is detected in 74.6% of patients with PTC and 47.7% with ATC in Japan (4). Therefore, molecular targeted drugs for BRAF are a rational therapeutic option for thyroid cancer with the *BRAF* V600E mutation (5, 6). In a Japanese phase 2 trial involving patients with *BRAF* V600E-mutated thyroid cancer, a BRAF inhibitor, encorafenib (ENC), plus a MEK inhibitor, binimetinib (BIN), demonstrated a clinically meaningful efficacy in terms of the primary endpoint of objective response rate (ORR; 54.5% in all patients (*n* = 22), 47.1% with DTC (*n* = 17), and 80.0% with ATC (*n* = 5)) in vascular endothelial growth factor receptor tyrosine kinase inhibitor (VEGFR-TKI)-refractory, intolerant, or ineligible settings (7). Additionally, the ENC + BIN regimen achieved a favorable duration of response (90.9%), progression-free survival (78.8%), and overall survival at 12 months (81.8%), with a manageable toxicity profile. Consequently, the use of ENC + BIN was approved in Japan for unresectable *BRAF*-mutated thyroid cancer that has progressed after chemotherapy and for *BRAF*-mutated unresectable ATC. Based on the outcomes of the study, the ENC + BIN regimen is also applicable as a first-line treatment for patients who are intolerant of or ineligible for VEGFR-TKIs.

Given the long lifespan expectancy due to the better prognosis of patients with thyroid cancer, especially DTC, compared to those with other cancer types, the health-related quality of life (HR-QoL) of patients is of concern. Survivors of thyroid cancer score significantly lower HR-QoL than the cancer-free group in functional and symptom scales (8). Patients with DTC treated with radioactive iodine (RAI) also have lower HR-QoL compared to the general cancer-free population (9). Although surgical resection can improve HR-QoL of patients with ATC to some extent (10), its rapidly progressing characteristics lead to generally poor HR-QoL outcomes. The effect of drug therapy on HR-QoL has been reported in patients with DTC, but not with ATC. In Japan, multitargeted tyrosine kinase inhibitors (MKIs), such as lenvatinib (LEN) and sorafenib, are the standard of care for RAI-refractory DTC that is not amenable to curative treatment. LEN can cause toxicities that have negative impacts on the HR-QoL (11). Correspondingly, LEN treatments are implemented with a dose reduction or interruption considering the balance between effectiveness and toxicities in the real-world setting (12, 13). Common toxicities induced by BRAF and MEK inhibitors are different from those induced by MKIs, leading to differing HR-QoL between these regimens, and only the MERAIODE trial shows the effect of toxicities by BRAF and MEK inhibitors on HR-QoL using the European Organization for Research and Treatment of Cancer Quality of Life Questionnaire (EORTC QLQ) – a core questionnaire of QLQ-C30 (7, 14, 15). Recently, EORTC QLQ-THY34, a thyroid cancer patient module, has been developed to fully capture thyroid cancer-specific issues (16). A further elucidation of how BRAF and MEK inhibition affects HR-QoL of patients with thyroid cancer using QLQ-THY34, as well as QLQ-C30, is warranted for treatment selection based on patient-reported outcomes.

In this study, we aimed to explore the effect of treatment with ENC + BIN on HR-QoL in patients with thyroid cancer, including DTC and ATC, using EORTC QLQ-C30 and QLQ-THY34.

## Materials and methods

### Study design and patients

This is a phase 2, open-label, uncontrolled, multicenter trial (Japan Registry of Clinical Trials registration No. jRCT2011200018) assessing the efficacy and safety of the combination of ENC + BIN in patients with *BRAF* V600E-mutated thyroid cancer (7). Patients orally received ENC (450 mg) once daily and BIN (45 mg) twice daily over a 28-day cycle until unacceptable toxicity, disease progression, or consent withdrawal. The primary endpoint, ORR, was met and has been previously reported in the primary publication (7), and this secondary analysis focuses on HR-QoL outcomes of patients in the trial, which were prespecified secondary endpoints.

Patients were enrolled between March 2021 and May 2022 and eligible to participate if they met all of the following inclusion criteria: age ≥20 years; histological diagnosis of locally advanced or distant metastatic thyroid cancer that was not amenable to curative treatment; *BRAF* V600E mutation in their tumor tissues or blood samples, confirmed with a central laboratory test; refractory to or intolerant of ≥1 oral VEGFR-targeted drugs for thyroid cancer or considered to be medically ineligible for those drugs; ≥1 measurable lesion by Response Evaluation Criteria in Solid Tumors (RECIST v1.1) (17) assessed at a local institution; Eastern Cooperative Oncology Group (ECOG) performance status (PS) of 0 or 1; a life expectancy of ≥3 months; and ability to swallow, ingest, and absorb oral drugs.

The Ethical Review Committee for Clinical Trials at Ono Pharmaceutical Co., Ltd., conducted the protocol review of this study on October 14, 2020, and approved the trial protocol on October 15, 2020. The trial protocol was also approved by the institutional review board at each site. This study was conducted in accordance with the Declaration of Helsinki and local regulations. All patients provided written informed consent.

### Questionnaires and data collection

HR-QoL was measured using the following two European Organization for Research and Treatment of Cancer Quality of Life Questionnaires (EORTC QLQs): EORTC QLQ-C30 (18) and EORTC QLQ-THY34 (16, 19). QLQ-C30 was developed to assess HR-QoL of cancer patients, which consists of global health status, functional scales, and symptom scales. QLQ-THY34 complements the QLQ-C30 by assessing HR-QoL issues specific to thyroid cancer patients, which consists of social support and symptom scales (see Supplementary Table 1 for details (see section on Supplementary materials given at the end of the article)). A commercial license for the use of these instruments was obtained from the EORTC Quality of Life Group in accordance with their guidelines.

HR-QoL surveys using these questionnaires were conducted during the screening period, at cycles 2 and 3, and subsequently at the time of tumor evaluation. The surveys were also performed at the end of study treatment and 28 days after the last study treatment (Supplementary Table 2). Scoring was performed only when both questionnaires were completed on the same date. Scores within the specified time windows during and after treatment were used based on actual visit dates, to include both scheduled and unscheduled visits. We define these time windows in Supplementary Table S3.

### Statistical analysis

The survey completion rate at each time point was calculated as the proportion of patients who completed the surveys among those who were able to take the survey (Supplementary Table 3). Patients were measured using the EORTC QLQ-C30 and QLQ-THY34, in comparison with the baseline score, which were then summarized as the mean score of all patients for each measure during the screening period. The minimal important change (MIC) threshold was set at 10 points, following previous studies (20, 21). We performed an exploratory statistical comparison between HR-QoL scores at baseline and at each time point using the Wilcoxon signed-rank test without multiple comparisons and set a nominal *P*-value less than 0.05 as the threshold.

## Results

### Study participants

At data cutoff (October 26, 2022), a total of 22 patients with *BRAF* V600E-mutated thyroid cancer (males: 12; median age: 68.0 years (range: 50–77)) were enrolled (see Table 1 for patients’ characteristics). Of all the patients, 17 had DTC and 5 had ATC. Among all patients, 14 (82.4%) received RAI therapy for DTC and 20 (90.9%) received one or more MKIs.

**Table 1:** Characteristics of patients.

Characteristics	Overall (*n* = 22)	DTC (*n* = 17)	ATC (*n* = 5)
Sex			
Male	12 (54.5%)	10 (58.8%)	2 (40.0%)
Female	10 (45.5%)	7 (41.2%)	3 (60.0%)
Age			
Median (range)	68.0 (50–77)	67.0 (50–75)	74.0 (60–77)
<65 years	9 (40.9%)	8 (47.1%)	1 (20.0%)
≥65 years	13 (59.1%)	9 (52.9%)	4 (80.0%)
ECOG PS			
0	6 (27.3%)	6 (35.3%)	0
1	16 (72.7%)	11 (64.7%)	5 (100%)
Prior surgery			
Yes	21 (95.5%)	16 (94.1%)	5 (100%)
No	1 (4.5%)	1 (5.9%)	0
Prior VEGFR-TKI			
0	2 (9.1%)	1 (5.9%)	1 (20.0%)
1	16 (72.7%)	12 (70.6%)	4 (80.0%)
≥2	4 (18.2%)	4 (23.5%)	0
Prior RAI therapy			
Yes	15 (68.2%)	14 (82.4%)	1 (20.0%)
No	7 (31.8%)	3 (17.6%)	4 (80.0%)
Metastasis lesion sites			
Lung	16 (72.7%)	12 (70.6%)	4 (80.0%)
Lymph nodes	16 (72.7%)	13 (76.5%)	3 (60.0%)
Bone	6 (27.3%)	4 (23.5%)	2 (40.0%)
Pleura	5 (22.7%)	4 (23.5%)	1 (20.0%)
Others	7 (31.8%)	5 (29.4%)	2 (40.0%)

ATC, anaplastic thyroid cancer; DTC, differentiated thyroid cancer; ECOG PS, Eastern Cooperative Oncology Group performance status; RAI, radioactive iodine; VEGFR, vascular endothelial growth factor receptor; TKI, tyrosine kinase inhibitor.

### HR-QoL of all patients

**Figure 1:**
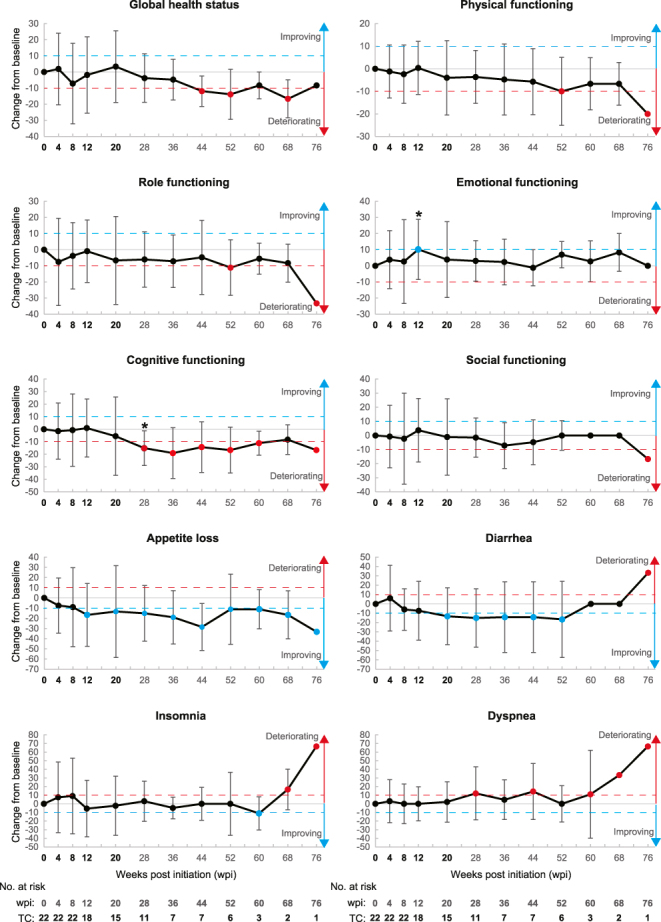
Empirical mean change from baseline over time for EORTC QLQ-C30 domains. Line plots showing empirical mean change from baseline over time regarding the indicated domains. Numbers at risk for all domains are presented at the bottom of each column. The blue and red dashed lines in each graph indicate differences of MIC for improvement and deterioration, respectively. *P < 0.05 (Wilcoxon signed-rank test).

**Figure 2:**
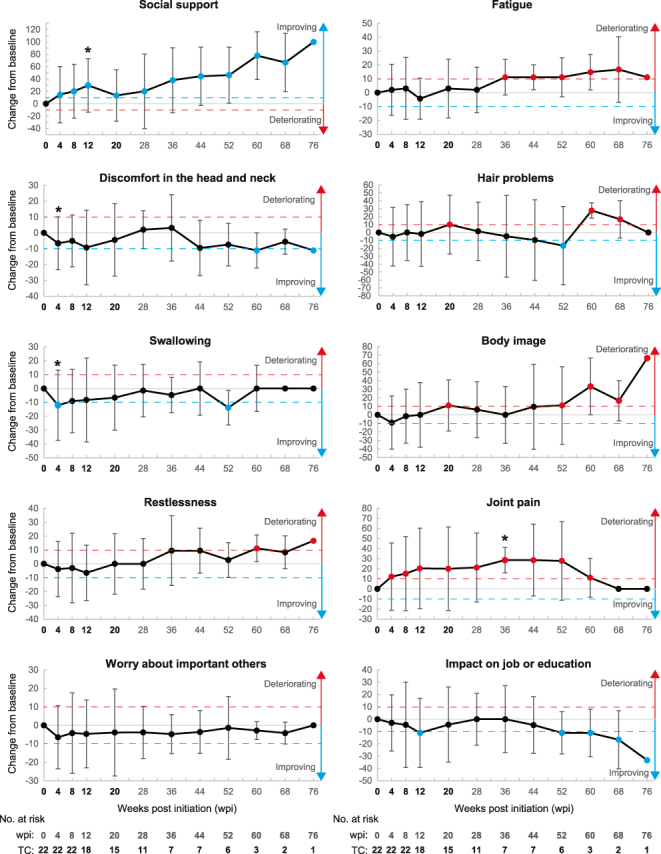
Empirical mean change from baseline over time for EORTC QLQ-THY34 domains. Line plots showing empirical mean change from baseline over time regarding the indicated domains. Numbers at risk for all domains are presented at the bottom of each column. The blue and red dashed lines in each graph indicate differences of MIC for improvement and deterioration, respectively. *P < 0.05 (Wilcoxon signed-rank test).

We analyzed the longitudinal changes in HR-QoL of patients with thyroid cancer during the study assessment. Completion rates for EORTC QLQ-C30 and QLQ-THY34 were 100% (22 of 22 patients) from treatment initiation to 8 weeks post-initiation (wpi), which gradually decreased to 68% (15 of 22) at 20 wpi and 50% (11 of 22) at 28 wpi (Supplementary Table S3). A reduction in completion rates at 28 wpi resulted from 9 treatment discontinuations (reasons: 6 progressive diseases (PDs), 1 AE, 1 consent withdrawal, and 1 censor) and 2 exclusions due to response outside the designated time period. Therefore, we first focused on the HR-QoL scores until 20 wpi. Patients generally maintained HR-QoL scores at their baseline level until 20 wpi. Patients showed longitudinal changes in HR-QoL less than MIC from baseline on most domains (29 of 32 domains, 90.6%), including global health status (Supplementary Tables S4 and S5). In the other 3 domains (9.4%), patients showed differences of MIC or more consecutively (improved direction: appetite loss and social support; deteriorated direction: joint pain). Although *P*-values were exploratory and unadjusted, statistical analyses showed that five time points differed by MIC or more from baseline with a *P*-value less than 0.05 (indicated by blue (improved direction: 4 time points) and red (deteriorated direction: 1 time point) circles with asterisks in Figs 1 and 2 and Supplementary Figs 1 and 2); however, these changes occurred sporadically rather than consistently (Supplementary Tables S4 and S5). In addition, patients’ scores related to fear improved in QLQ-THY34 at 4 wpi (value: −8.59, *P*-value: 0.048) and 8 wpi (value: −9.6, *P*-value: 0.002) consecutively with *P*-values less than 0.05, although the differences from baseline were less than the MIC (indicated by a black circle with an asterisk in Supplementary Fig. 2).

Then, we conducted analyses of the entire follow-up period, focusing on HR-QoL tendencies toward improvement and deterioration, which were defined by differences of MIC or more consecutively in either direction. Patients showed tendency toward either direction on 7 domains in QLQ-C30 and 6 domains in QLQ-THY34; there were two improvements (appetite loss and diarrhea) and five deteriorations (global health status, cognitive functioning, fatigue, dyspnea, and insomnia) in QLQ-C30 (Fig. 1 and Supplementary Fig. 1 and Table S4) and two improvements (social support and impact on job or education) and four deteriorations (fatigue, hair problems, body image, and joint pain) in QLQ-THY34 (Fig. 2 and Supplementary Fig. 2 and Table S5). Specifically, patients showed deteriorations consecutively in HR-QoL scores for joint pain in QLQ-THY34 from 4 wpi until 60 wpi (Fig. 2, 4th row on the right, and Supplementary Table S5).

### HR-QoL of patients with DTC and ATC

We then analyzed the HR-QoL scores by histological type, DTC and ATC. At baseline, patients with DTC had better mean scores than those with ATC regarding global health status (70.10 for patients with DTC and 53.33 for patients with ATC) (Supplementary Table 4). Consistently, patients with DTC had a better mean score than those with ATC in several domains, such as constipation in QLQ-C30 (Supplementary Table S4) and discomfort in the head and neck, swallowing, dry mouth, and body image in QLQ-THY34 (Supplementary Table S5).

**Figure 3:**
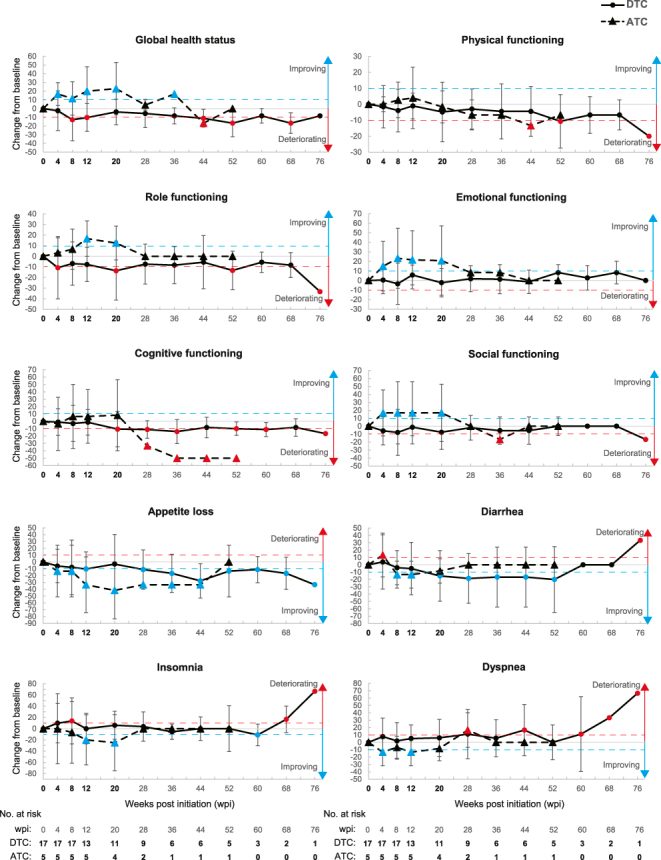
Empirical mean change from baseline over time by DTC and ATC for EORTC QLQ-C30 domains. Line plots showing empirical mean change from baseline over time by DTC (solid line) and ATC (dashed line) regarding the indicated domains. Numbers at risk for all domains are presented at the bottom of each column. The blue and red dashed lines in each graph indicate differences of MIC for improvement and deterioration, respectively.

**Figure 4:**
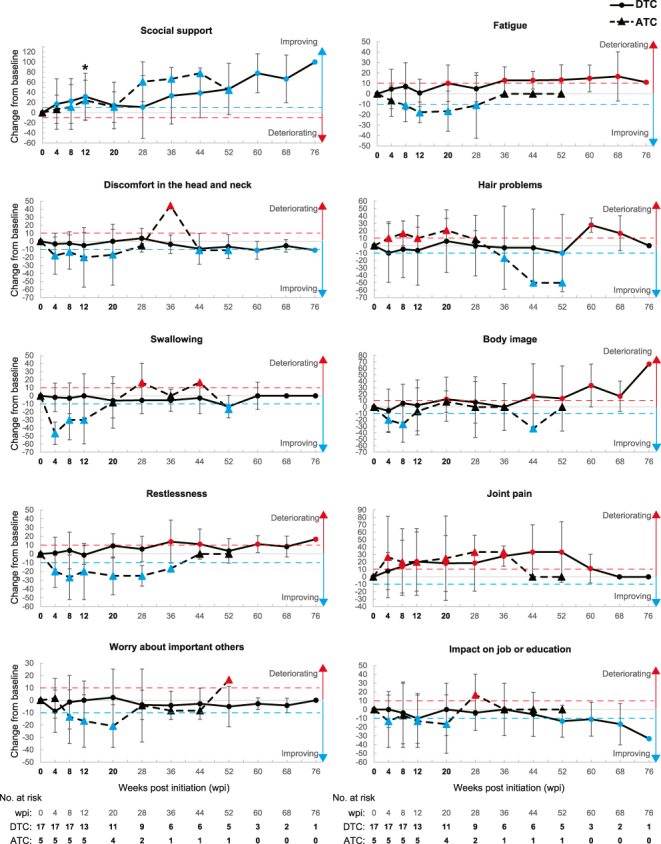
Empirical mean change from baseline over time by DTC and ATC for EORTC QLQ-THY34 domains. Line plots showing empirical mean change from baseline over time by DTC (solid line) and ATC (dashed) regarding the indicated domains. Numbers at risk for all domains are presented at the bottom of each column. The blue and red dashed lines in each graph indicate differences of MIC for improvement and deterioration, respectively. *P < 0.05 (Wilcoxon signed-rank test). All asterisks indicated in the figure are for scores of patients with DTC.

We observed no HR-QoL domains that consecutively differed by MIC or more from baseline with a *P*-value less than 0.05 during the study period in patients with either DTC or ATC (please note that *P*-values were exploratory and unadjusted). However, patients with DTC or ATC had tendencies toward improvements and deteriorations in some domains. Patients with ATC had tendencies toward improvement and deterioration in more domains than those with DTC: 15 domains for patients with DTC and 27 with ATC (Fig. 3 and Supplementary Fig. 3 and Table S4 for QLQ-C30 and Fig. 4 and Supplementary Fig. 4 and Table S5 for QLQ-THY34). Consistent with the results of all 22 patients, those with DTC and ATC showed persistent tendency toward deterioration in HR-QoL scores related to joint pain, one of the relatively frequent adverse reactions of ENC + BIN (7), and persisted 8–60 wpi for patients with DTC and 4–36 wpi with ATC (Fig. 4, 4th row on the right, and Supplementary Table S5). Tendencies toward either improvement or deterioration in HR-QoL issues occurred earlier in patients with ATC than those with DTC (Fig. 3 and Supplementary Fig. 3 and Table S4 for QLQ-C30 and Fig. 4 and Supplementary Fig. 4 and Table S5 for QLQ-THY34). Specifically, differences of MIC or more consecutively by 20 wpi were observed in 3 domains for patients with DTC and in 19 domains for those with ATC (improvements vs deteriorations were 1 vs 2 for DTC and 16 vs 3 for ATC). Particularly, patients with ATC showed a tendency toward improvement from the earliest assessment at 4 wpi in 4 domains in QLQ-THY34: discomfort in the head and neck, swallowing, body image, and restlessness. When evaluating HR-QoL scores individually, each patient with ATC improved scores by MIC or more from baseline at the first assessment after treatment initiation on swallowing (Supplementary Fig. 5).

## Discussion

This study investigated longitudinal changes in HR-QoL of patients with thyroid cancer treated with ENC + BIN using the EORTC QLQ-C30 and EORTC QLQ-THY34 questionnaires. Patients generally maintained stable HR-QoL during treatment with ENC + BIN; however, mean changes from baseline reached or exceeded MIC with a *P*-value less than 0.05 in certain domains at several time points: four improvements and three deteriorations. However, during the survey period, no differences of MIC or more with a *P*-value less than 0.05 were observed consecutively in either the positive or negative directions in scores for any domain in either questionnaire. Thus, our results suggest that the combination treatment with ENC + BIN generally maintained a stable HR-QoL during the treatment for patients with *BRAF* V600E-mutated thyroid cancer.

The COLUMBUS trial shows that the improved efficacy of the ENC + BIN regimen provides a positive effect on patient-reported outcomes: patients with BRAF-mutated melanoma maintain HR-QoL for an extended duration (22). Similarly, in this study, the ENC + BIN regimen demonstrated generally sustained HR-QoL in the global health and all functional scales, as well as most symptom scales, except for joint pain, in patients with thyroid cancer using both EORTC QLQ-C30 and EORTC THY-34 questionnaires. Therefore, patients with thyroid cancer generally maintain HR-QoL, including thyroid-specific symptoms, when treated with the ENC + BIN regimen. In the MERAIODE trial, the DAB + TRA regimen demonstrated continuous deterioration (MIC of 5 or more) in the global health and all the functional scales, as well as in some symptom scales, such as appetite loss and fatigue, in patients with DTC, using the EORTC QLQ-C30 but not EORTC THY-34 questionnaire (15). The difference between these two regimens may result from the difference in the duration of the trials (median follow-up was 11.5 months in this study vs 34 months in the MERAIODE trial) and MIC (10 vs 5). In addition, the sample size of these trials is limited, and one should interpret these differences with special caution.

Adverse events (AEs) with ENC + BIN in patients with melanoma were likely to occur within 24 weeks in the COLUMBUS study (23) and within 17 weeks in a Japanese real-world clinical setting (24). The current study was able to evaluate HR-QoL of more than half of the patients with either DTC or ATC until 20 weeks after treatment initiation, although we should interpret the scores cautiously beyond this point, as only half or fewer patients completed the questionnaires after 28 wpi. Therefore, this study period would be sufficient to assess the effects of AEs on HR-QoL.

Toxicities by treatment may negatively impact the HR-QoL. LEN treatment has been reported to cause frequent toxicities, including fatigue and dyspnea, that negatively impact the HR-QoL scores (11, 14). Notably, in this study, the HR-QoL scores of joint pain showed a sustained tendency toward deterioration in patients with thyroid cancer, including both patients with DTC and ATC. Arthralgia was one of the reported AEs in this trial (observed in 6 of 22 patients (27%)) (7) and is reported with a relatively high frequency in the treatment of melanoma using combinations of BRAF and MEK inhibitors (25, 26, 27). Therefore, it is regarded as one of the class effects of BRAF inhibitor therapies. Arthralgia can be managed with nonsteroidal anti-inflammatory drugs (NSAIDs) or by adjusting the dosage of BRAF and MEK inhibitors (28). It is important to implement proper management, such as NSAID treatment or dosage adjustment, considering the balance between the effectiveness and the patient’s HR-QoL. Moreover, our study showed that the QLQ-THY34 questionnaire captured symptoms of joint pain during the trial treatment period, suggesting that this questionnaire accurately assesses symptomatic adverse events, one of the key target patient-related outcomes by HR-QoL instruments (29), during treatment.

Even slowly progressing DTC can impair HR-QoL (8, 9), suggesting that more rapidly progressing ATC might have more severe outcomes. Accordingly, patients with ATC appeared to show worse HR-QoL than those with DTC on several domains at baseline. Among these, four domains (appetite loss, voice concerns, swallowing, and discomfort in the head and neck) might be associated with primary thyroid cancer and its spread to nearby tissues, including lymph nodes (2). In this trial, the ORR for ENC + BIN in the patients with ATC was 80%, with tumor shrinkage observed in all the patients (7). Notably, patients with ATC tended to improve HR-QoL scores on appetite loss, swallowing, and discomfort in the head and neck by MIC or more in the early treatment phase. These results suggest that ENC + BIN may have the potential to swiftly improve HR-QoL, possibly as a result of tumor response to treatment. Considering the small sample size (only 5 patients with ATC) and the analysis being exploratory, this interpretation should be generalized with special caution. Further studies with larger cohorts are warranted to evaluate this preliminary interpretation.

Based on the HR-QoL, efficacy, and safety of ENC + BIN in this trial, the therapy regimen can be a potential second-line therapy for patients with unresectable locally advanced or metastatic *BRAF* V600E-mutated thyroid cancer who discontinue standard therapies, such as LEN treatment, due to severe toxicities. The emergence of molecular target drugs, such as inhibitors of BRAF, MEK, RET, and NTRK, has broadened treatment options for patients with RAI-resistant DTC. Because patients with thyroid cancer, especially those with DTC, often require long-term drug treatment, it is important to explore treatment sequences that achieve a favorable balance between the effectiveness of treatment and patient HR-QoL in the future.

One of the limitations is that this study is an exploratory analysis of HR-QoL, with significance testing specified after protocol fixation. Additionally, the sample size was limited to 22 in this trial, and patients who completed the questionnaires at 28 wpi decreased to 50%, primarily due to discontinuation caused by PD; the sample size of patients with ATC was only 5. Because an outlier could substantially affect HR-QoL after 28 wpi or that of patients with ATC, the mean HR-QoL score difference from baseline under these circumstances should be interpreted with caution. Another limitation is the lack of a comparator group because this is a single-arm trial; therefore, the effect of ENC + BIN on HR-QoL cannot be compared to those of other treatments.

Treatment with ENC + BIN was generally associated with the maintenance of HR-QoL in patients with unresectable locally advanced or metastatic *BRAF* V600E-mutated thyroid cancer. These results further support the use of the treatment for patients with unresectable locally advanced or metastatic *BRAF* V600E-mutated thyroid cancer.

## Supplementary materials



## Declaration of interest

NK received a research grant from Ono Pharmaceutical, AbbVie, Boehringer Ingelheim, GlaxoSmithKline (GSK), and Bayer and honoraria for lectures from Bayer, Eisai, Ono Pharmaceutical, Eli Lilly, Novartis, Merck Biopharma, and MSD. MT received grants from Ono Pharmaceutical and Bayer; consulting fees from Boehringer Ingelheim, Janssen Pharmaceutical, Genmab, AbbVie, and Astellas; and payment for lectures from Ono Pharmaceutical, Merck Sharp & Dohme (MSD), Bayer, Rakuten Medical, Bristol-Myers Squibb (BMS), Merck Biopharma, Eisai, Eli Lilly, and Novartis. MT participated in an advisory board of MSD, Bayer, BMS, Eisai, AstraZeneca, Merus, Merck Biopharma, Pfizer, Eli Lilly, Boehringer Ingelheim, GSK, and Johnson & Johnson. STak received grants and honoraria from Daiichi-Sankyo, Novartis, Chugai Pharma, BMS, Eisai, MSD, AstraZeneca, and Taiho Pharmaceutical and an honorarium from Ono Pharmaceutical. STam has received honoraria for a speaker from Bayer Pharma Japan, Chugai, Eli Lilly Japan, Daiichi-Sankyo, Ono Pharmaceutical, and MSD KK. YS received a research grant from MSD and honoraria for lectures from Ono Pharmaceutical, Eisai, Eli Lilly, MSD, Bristol-Myers Squibb, and Merck Biopharma. SK received grants from Ono Pharmaceutical, Taiho Pharmaceutical, MSD, Nobelpharma, Janssen Pharmaceutical KK, BMS, Eli Lilly, Chugai, Daiichi-Sankyo, AstraZeneca, AbbVie, Kyowa Kirin, Astellas, Novartis, and Bayer and honoraria from Ono Pharmaceutical, Taiho Pharmaceutical, MSD, Daiichi-Sankyo, Merck KGaA, BMS, Eli Lilly, Chugai, Bayer, Eisai, Novartis, and Astellas. KI received payment for lectures from Ono Pharmaceutical, Eli Lilly, and Novartis. YH and SU are employed by Ono Pharmaceutical. IS received grants from Bayer and Eisai for his institution; received honoraria for lectures and travel expenses from Eli Lilly, Eisai, Ono Pharmaceutical, and Novartis to attend a meeting; and participated in an advisory board of Takeda and Eli Lilly. HI and AN declare no competing interests.

## Funding

This study was funded by Ono Pharmaceutical Co., Ltd.

## Author contribution statement

NK and MT contributed to conception and design of the study and acquisition and interpretation of data; HI, STak, AN, STam., YS, SK, and KI contributed to acquisition of data; YH contributed to conception and design of the study, interpretation of data, and drafting; SU contributed to design of the study, analysis and interpretation of data, and drafting; and IS contributed to conception and design of the study and acquisition and interpretation of data. All authors contributed to the revision of the article, approved the final version, and had final responsibility for the decision to submit for publication.
