# Supplementary material for: Health-related quality of life with encorafenib plus binimetinib for BRAFV600E thyroid cancer
Source: Eur Thyroid J. 2026 Apr 3;15(2):ETJ250273. doi: 10.1530/ETJ-25-0273 (PMC13052824; doi:10.1530/ETJ-25-0273)
Supplement: Supplementary file 1 [file supplementary_materials.pdf]

Supplementary Figure 1: Empirical means change from baseline over time for EORTC QLQ-C30 domains: other domains (related to Figure 1)

Line plots showing empirical mean change from baseline over time regarding indicated domains. Numbers at risk for all domains are presented at the bottom of each column. Blue and red dashed lines in each graph indicate differences of MIC for improvement and deterioration, respectively. *, $p < 0.05$ (Wilcoxon signed-rank test).

Supplementary Figure 1

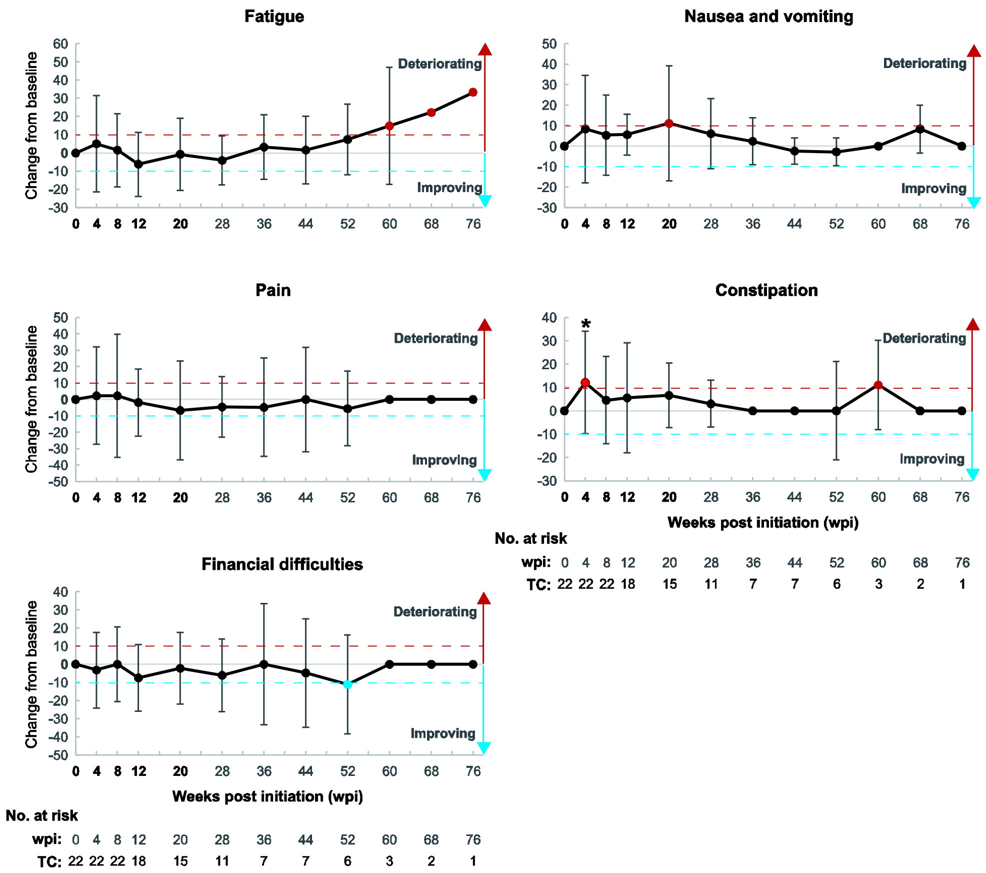

Supplementary Figure 2: Empirical mean change from baseline over time for EORTC QLQ-THY34 domains: other domains (related to Figure 2)

Line plots showing empirical mean change from baseline over time regarding indicated domains. Numbers at risk for all domains are presented at the bottom of each column. Blue and red dashed lines in each graph indicate differences of MIC for improvement and deterioration, respectively. *, $p < 0.05$ (Wilcoxon signed-rank test).

Supplementary Figure 2

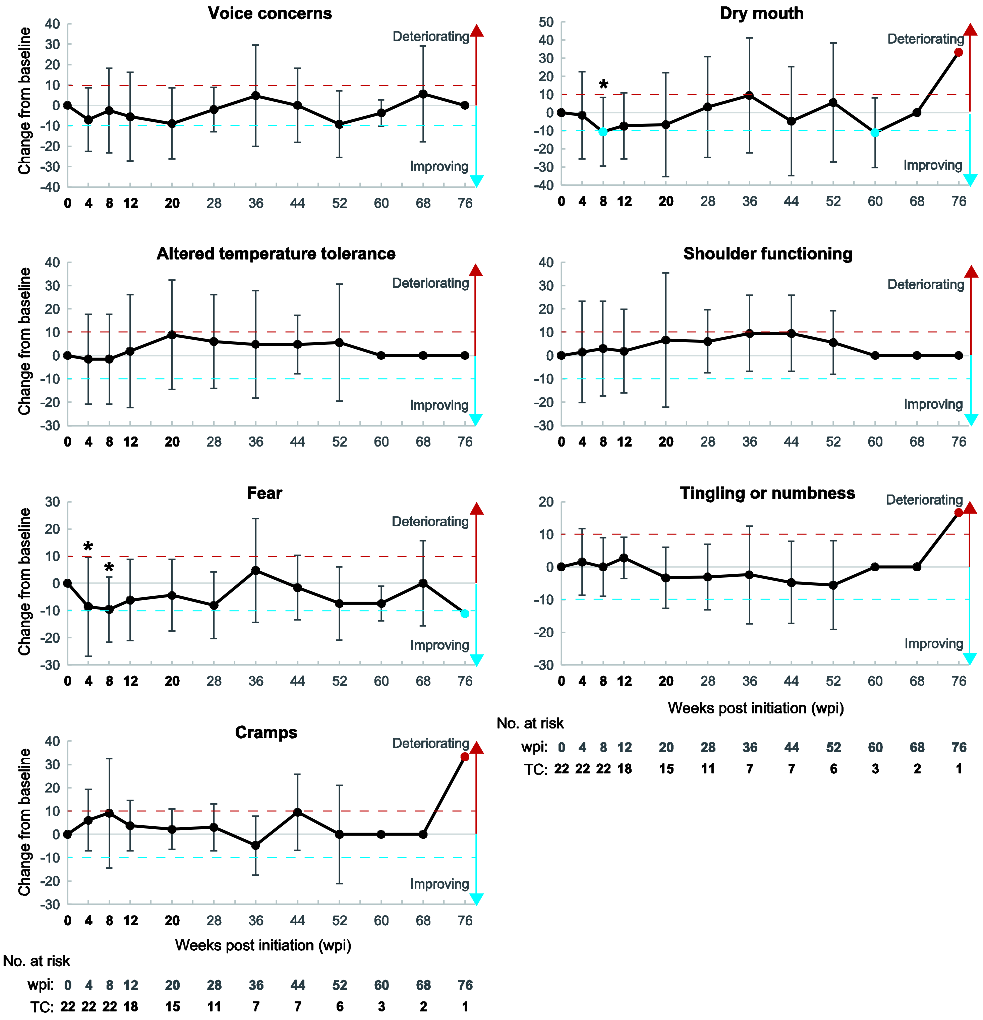

Supplementary Figure 3: Empirical means change from baseline over time by DTC and ATC for EORTC QLQ-C30 domains: other domains (related to Figure 3)

Line plots showing empirical mean change from baseline over time by DTC (solid line with circles) and ATC (dashed line with triangles) regarding indicated domain. Numbers at risk for all domains are presented at the bottom of each column. Blue and red dashed lines in each graph indicate differences of MIC for improvement and deterioration, respectively.

Supplementary Figure 3

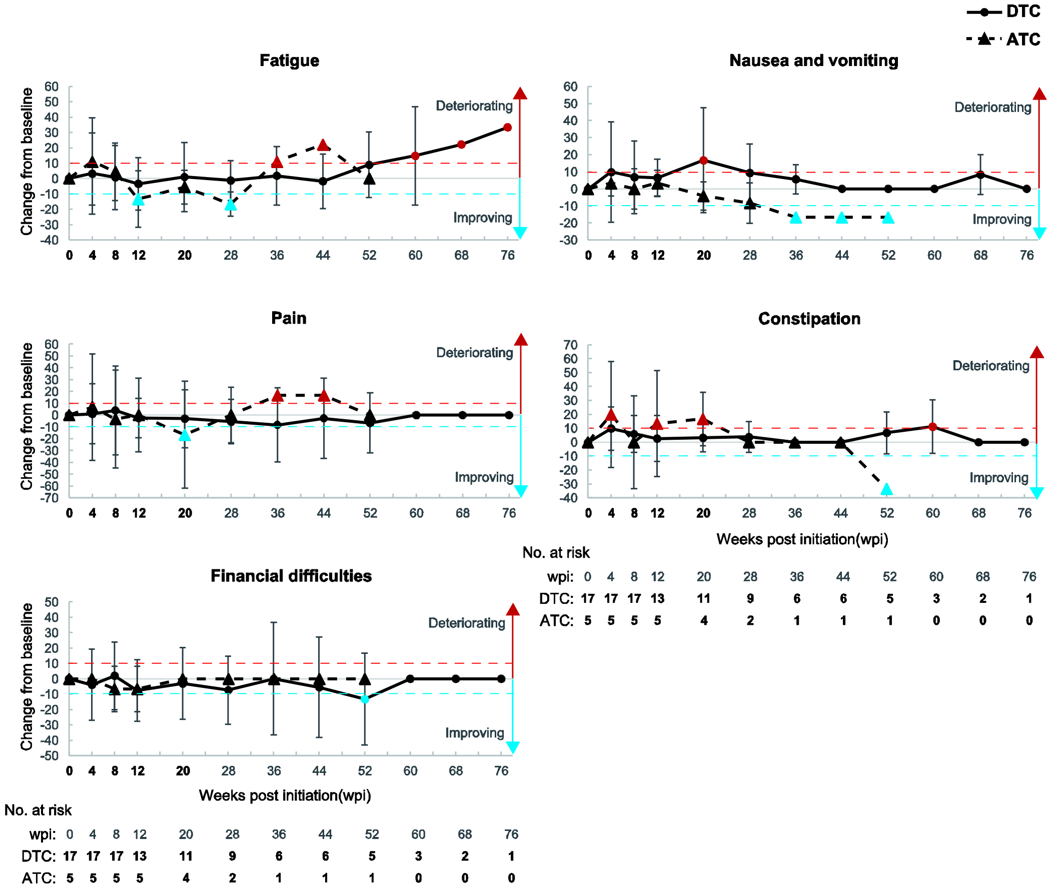

Supplementary Figure 4: Empirical means change from baseline of each patient with ATC over time for EORTC QLQ-THY34 domains: other domains (related to Figure 4)

Line plots showing empirical mean change from baseline over time by DTC (solid line with circles) and ATC (dashed line with triangle) regarding indicated domains. Numbers at risk for all domains are presented at the bottom of each column. Blue and red dashed lines in each graph indicate differences of MIC for improvement and deterioration, respectively. *, $p < 0.05$ (Wilcoxon signed-rank test). All * indicated in the figure are for scores of patients with DTC.

Supplementary Figure 4

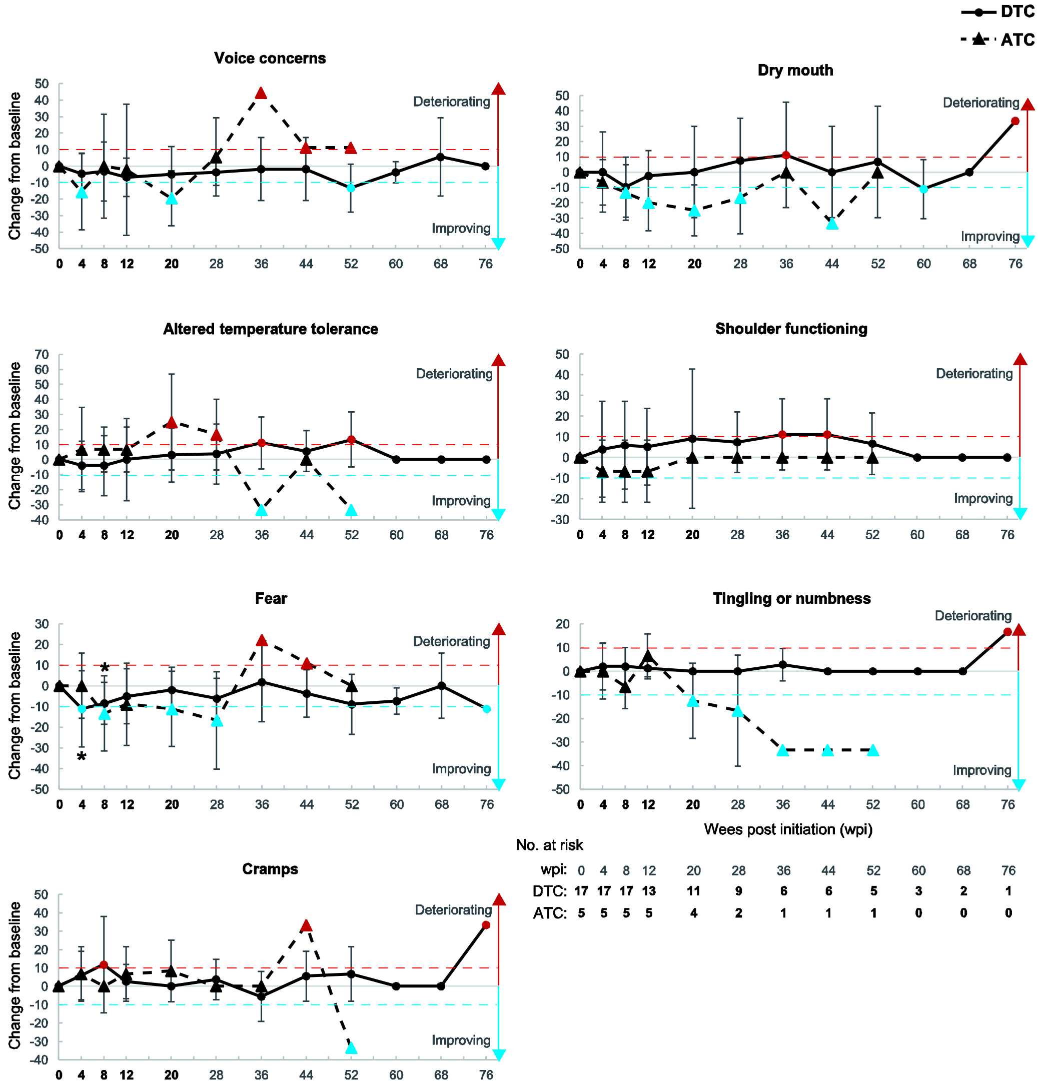

No. at risk

wpi:	0	4	8	12	20	28	36	44	52	60	68	76
DTC:	17	17	17	13	11	9	6	6	5	3	2	1
ATC:	5	5	5	5	4	2	1	1	1	0	0	0

No. at risk

wpi:	0	4	8	12	20	28	36	44	52	60	68	76
DTC:	17	17	17	13	11	9	6	6	5	3	2	1
ATC:	5	5	5	5	4	2	1	1	1	0	0	0

Supplementary Figure 5: Empirical change from baseline of each patient with ATC overtime for EORTC QLQ-THY34 domains

Line plots showing empirical mean change from baseline over time regarding indicated domains.

Supplementary Figure 5

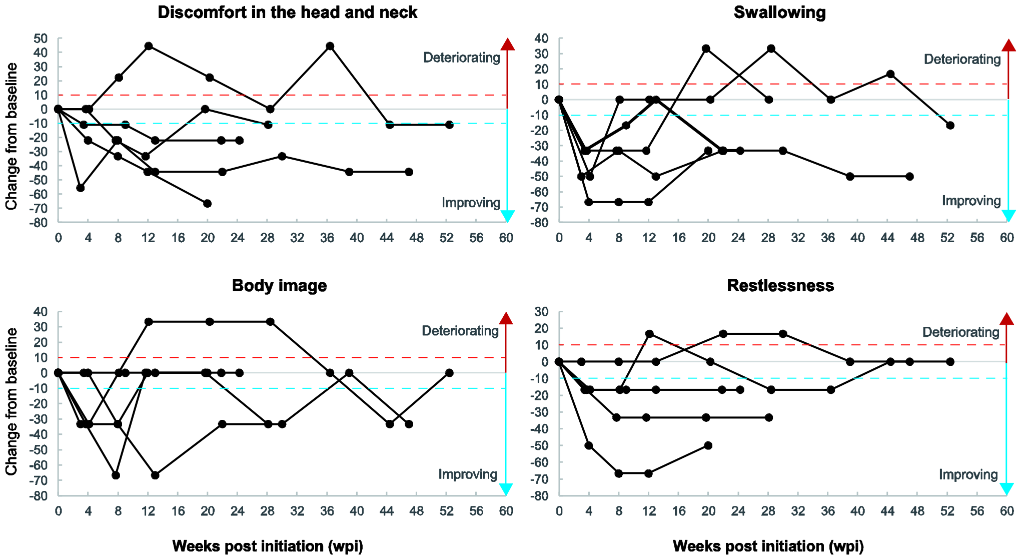

Supplementary Table 1: Questionnaires for HR-QoL Assessments

	**EORTC QLQ-C30**	**EORTC QLQ-THY34**
Evaluation points	Cancer-related QoL	Thyroid cancer-related QoL
Score	0 ~ 100	0 ~ 100
Components	Global health status<br>Functional scales (5 domains*)<br>Symptom scales (9 domains†)	Social support (functional scale)<br>Symptom scales (16 domains ‡)

*, 5 domains are Physical functioning, Role functioning, Emotional functioning, Cognitive functioning, Social functioning; †, 9 domains are Fatigue, Nausea and vomiting, Pain, Dyspnea, Insomnia, Appetite loss, Constipation, Diarrhea, Financial difficulties; ‡, Discomfort in the head and neck, Fatigue, Fear, Hair problems, Restlessness, Swallowing, Worry about important others, Tingling or numbness, Voice concerns, Body image, Cramps, Dry mouth, Altered temperature tolerance, Impact on job or education, Joint pain, Shoulder functioning. For global health status and functional scales, the higher the score, the better HR-QoL. For symptom scales, the lower the score, the better HR-QoL.

Supplementary Table 2: HR-QoL assessment schedule for patients with TC.

	Screening period		Study treatment period			[illegible]
Visit date		cycle2	cycle3	cycle4 and later	End of the study treatment period	[illegible]
	-30 to -1	-7 to 0	-7 to 0	-7 to 0	+7 from the Day on which the End of the Study Treatment Period was Decided	[illegible]
HR-QoL †	Mandatory	Mandatory	Mandatory	Voluntary ‡	Mandatory	[illegible]

†, EORTC QLQ-C30 and QLQ-THY34 were performed. All assessments were to be performed before the study proce[illegible]
The survey was performed at the time of tumor evaluations. However, it was voluntary at the time of an optional tumor [illegible]
was to be performed only if a tumor evaluation was performed.

Supplementary Table 3: Survey collection rate at each time point.

**Time of assessment**	**Time window**	**Number of patients (%)**<br>**Overall**	**Number of patients (%)**<br>**DTC**
Baseline	during screening	22 (100)	17 (100)
4 wpi	from day 2 to 42	22 (100)	17 (100)
8 wpi	from day 43 to 70	22 (100)	17 (100)
12 wpi	from day 71 to 98	18 (82)	13 (76)
20 wpi	from day 127 to 154	15 (68)	11 (65)
28 wpi	from day 183 to 210	11 (50)	9 (53)
36 wpi	from day 239 to 266	7 (32)	6 (35)
44 wpi	from day 295 to 322	7 (32)	6 (35)
52 wpi	from day 351 to 378	6 (27)	5 (29)
60 wpi	from day 407 to 434	3 (14)	3 (18)
68 wpi	from day 463 to 490	2 (9)	2 (12)
76 wpi	from day 519 to 546	1 (5)	1(6)

Supplementary Table 4: HR-QoL scores at baseline and improvements and deteriorations over time of patients with TC, DTC, or ATC assessed by EORTC QLQ-C30.

QLQ-C30	Cancer type	Mean score (SD) in each domain at baseline	Time points whose changes from baseline were more than MIC											
			0	4	8	12	20	28	36	44	52	60	68	76
Global health status *	TC	66.29 (22.04)								Red	Red		Red	
	DTC	70.10 (17.94)			Red	Red				Red	Red		Red	
	ATC	53.33 (31.51)		Blue	Blue	Blue	Blue		Blue	Red				
Physical functioning †	TC	84.85 (14.57)									Red			Red
	DTC	86.27 (14.62)									Red			Red
	ATC	80.00 (14.91)								Red				
Role functioning †	TC	84.85 (21.15)									Red			Red
	DTC	87.25 (22.46)		Red			Red				Red			Red
	ATC	76.67 (14.91)				Blue	Blue							
Emotional functioning †	TC	84.09 (18.35)				Blue								
	DTC	87.75 (10.26)												
	ATC	71.67 (33.12)		Blue	Blue	Blue	Blue							
Cognitive functioning †	TC	81.82 (19.18)						Red	Red	Red	Red	Red		Red
	DTC	84.31 (13.78)					Red	Red	Red		Red	Red		Red
	ATC	73.33 (32.49)						Red	Red	Red	Red			
Social functioning †	TC	84.85 (25.15)												Red
	DTC	87.25 (18.19)												Red
	ATC	76.67 (43.46)		Blue	Blue	Blue	Blue		Red					
Fatigue ‡	TC	33.33 (20.57)										Red	Red	Red
	DTC	31.37 (21.60)										Red	Red	Red
	ATC	40.00 (16.85)				Blue		Blue	Red	Red				

Nausea and vomiting ‡	TC	3.03 (11.07)
	DTC	2.94 (12.13)
	ATC	3.33 (7.45)
Pain ‡	TC	25.00 (29.88)
	DTC	25.49 (29.53)
	ATC	23.33 (34.56)
Dyspnoea ‡	TC	24.24 (23.42)
	DTC	23.53 (25.72)
	ATC	26.67 (14.91)
Insomnia ‡	TC	22.73 (29.79)
	DTC	19.61 (26.51)
	ATC	33.33 (40.82)
Appetite loss ‡	TC	27.27 (30.23)
	DTC	23.53 (28.30)
	ATC	40.00 (36.51)
Constipation ‡	TC	10.61 (23.87)
	DTC	5.88 (13.10)
	ATC	26.67 (43.46)
Diarrhoea ‡	TC	12.12 (24.22)
	DTC	11.76 (26.20)
	ATC	13.33 (18.26)
Financial difficulties ‡	TC	12.12 (19.37)
	DTC	13.73 (20.61)
	ATC	6.67 (14.91)

*, Global health status (the higher the score, the better HR-QoL). †, Functional scales (the higher the score, the better HR-QoL). ‡, Symptom scales (the lower the score, the better HR-QoL).

White: baseline. Gray: change less than the minimal important change (MIC). Blue: change of MIC or more in a positive direction (improvement). Red, change of MIC or more in a negative direction.

Abbreviations: ATC, anaplastic thyroid cancer; DTC, differentiated thyroid cancer; SD, standard deviation; TC, thyroid cancer.

Supplementary Table 5: HR-QoL scores at baseline and improvements and deteriorations over time of patients with TC, DTC, or ATC assessed by EORTC QLQ-THY34.

QLQ-THY34	Cancer type	Mean score (SD) in each domain at baseline
Social support *	TC	61.62 (37.99)
	DTC	60.78 (38.51)
	ATC	64.44 (40.37)
Fatigue †	TC	18.69 (17.28)
	DTC	15.03 (14.67)
	ATC	31.11 (21.37)
Discomfort in the head and neck †	TC	25.76 (23.10)
	DTC	18.30 (14.67)
	ATC	51.11 (30.02)
Voice concerns †	TC	25.25 (18.20)
	DTC	21.57 (14.42)
	ATC	37.78 (25.58)
Hair problems †	TC	27.27 (35.09)
	DTC	26.47 (34.39)
	ATC	30.00 (41.50)
Swallowing †	TC	28.79 (26.82)
	DTC	20.59 (20.86)
	ATC	56.67 (27.89)
Dry mouth †	TC	27.27 (26.50)
	DTC	21.57 (20.21)

Time points whose changes from baseline were more than MIC

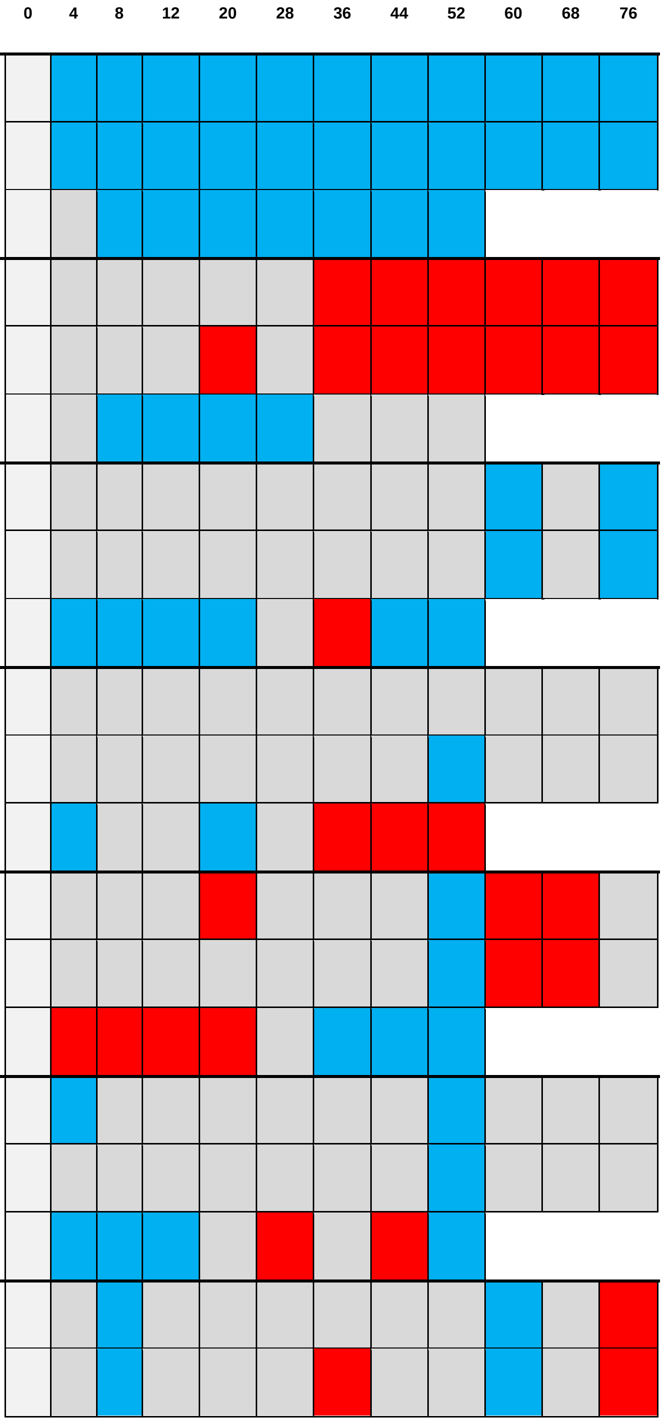

	ATC	46.67 (38.01)
Altered temperature tolerance †	TC	13.64 (16.77)
	DTC	13.73 (16.91)
	ATC	13.33 (18.26)
Body image †	TC	33.33 (32.53)
	DTC	25.49 (30.11)
	ATC	60.00 (27.89)
Restlessness †	TC	15.91 (20.23)
	DTC	11.76 (14.15)
	ATC	30.00 (32.06)
Shoulder functioning †	TC	7.58 (17.61)
	DTC	5.88 (13.10)
	ATC	13.33 (29.81)
Fear †	TC	28.79 (14.41)
	DTC	26.14 (14.67)
	ATC	37.78 (9.94)
Joint pain †	TC	12.12 (19.37)
	DTC	11.76 (20.21)
	ATC	13.33 (18.26)
Tingling or numbness †	TC	3.03 (8.35)
	DTC	0.98 (4.04)
	ATC	10.00 (14.91)
Cramps †	TC	6.06 (16.70)
	DTC	3.92 (11.07)
	ATC	13.33 (29.81)
Worry about important others †	TC	26.14 (19.30)
	DTC	25.00 (16.93)

	ATC	30.00 (28.01)
Impact on job or education †	TC	22.73 (29.79)
	DTC	23.53 (25.72)
	ATC	20.00 (44.72)

*, Social support (the higher the score, the better HR-QoL). †, Symptom scales (the lower the score, the better HR-QoL).

White: baseline. Gray: change less than the minimal important change (MIC). Blue: change more of MIC or more in a positive direction (improvement). Red: change more of MIC or more in a negative direction (deterioration).

Abbreviations: ATC, anaplastic thyroid cancer; DTC, differentiated thyroid cancer; SD, standard deviation; TC, thyroid cancer
